# Content of Serious Illness Care conversation documentation is associated with goals of care orders—a quantitative evaluation in hospital

**DOI:** 10.1186/s12904-022-01006-2

**Published:** 2022-06-29

**Authors:** Seema King, Maureen Douglas, Sidra Javed, Jocelyn Semenchuk, Sunita Ghosh, Fiona Dunne, Aliza Moledina, Konrad Fassbender, Jessica Simon

**Affiliations:** 1 Department of Community Health Sciences, University of Calgary, Calgary, AB Canada; 2 Covenant Health Palliative Care Institute, Edmonton, AB Canada; 3 Department of Medicine, University of Calgary, Calgary, AB Canada; 4 Alberta Health Services, Calgary Zone, Calgary, AB Canada; 5 University of Alberta, Edmonton, AB Canada; 6 Department of Medicine, The Ottawa Hospital, Ottawa, ON Canada; 7 Department of Oncology, University of Alberta, Edmonton, AB Canada; 8 Department of Oncology, Division of Palliative Medicine, University of Calgary, AB T2N 4Z6 Calgary, Canada

**Keywords:** Advance care planning, Serious illness, Goals of care, Hospitals, Communication

## Abstract

**Background:**

The Serious Illness Care Program (SICP) increases quality of documentation about patients’ values and priorities, but it is not known whether patient characteristics and goals of care are associated with the elements documented. The purpose of this study was to explore for associations between the quantity and type of elements documented after SICP conversations with patient characteristics and goals of care order.

**Methods:**

Documentation of SICP conversations by internal medicine physicians with hospitalized patients was evaluated in a retrospective chart review between March 2018 to December 2019. The conversations occurred after SICP implementation in a Tertiary Hospital, Medical teaching unit which uses “Goals of Care Designation” (GCD) medical orders to communicate a patient’s general intent, specific interventions, and preferred locations of care. A validated SICP codebook was used to determine the frequency of conversation elements documented for (1) Goals and Values; (2) Prognosis/illness understanding; (3) End-of-life care planning and (4) GCD/Life-sustaining treatment preferences. Univariate and multivariate generalized linear models were used to analyze associations between quantity of elements documented and patient characteristics (age, gender, frailty, language spoken and GCD).

**Results:**

Of 175 SICP conversations documented, in the univariate analysis more goals and values were documented for patients who understand/speak English (0.89; 95% CI: 0.14 - 1.63) and more content was recorded for patients with a non-resuscitative GCD focus (“Medical”: 2.42; 95% CI: 1.51 – 3.33; “Comfort”: 1.06; 95% CI: 0.24 – 1.88) although not in all domains. In the multivariate analysis, controlling for age, gender, language and frailty, the association between content scores and GCD remained highly significant. Patients with a non-resuscitative GCD had higher total domain scores than those with a resuscitative GCD (“Medical”: 1.27 95% CI: 0.42–2.13; “Comfort”: 2.67, 95% CI:1.71–3.62)**.**

**Conclusion:**

The type of content documented by physicians after a SICP conversation is associated with the patient’s goals of care.

**Supplementary Information:**

The online version contains supplementary material available at 10.1186/s12904-022-01006-2.

## Background

Patients with serious illnesses benefit from meaningful communication about their priorities and goals with healthcare providers [1, 2]. Conversations eliciting patients’ values inform shared medical decision-making processes and guide both current and future care planning [3]. System changes to encourage and document this communication could help address the alarming discordance between hospitalized patients’ preferences and their medical orders [4] and enables sharing of key information between healthcare providers across different patient encounters. Conversation documents complement legal documents (such as advance directives) and medical orders (such as “Do not attempt resuscitation”) and when patient capacity is impaired these documents can facilitate patient-centered decision-making by surrogates and healthcare providers.

The Serious Illness Care Program (SICP) [5] demonstrated that routinizing and improving the quality of clinician-patient communication enhances patient outcomes, decreasing anxiety and depression [6]. Randomized controlled trials of the SICP in oncology [7] and primary care [8] also described positive impacts on the quality and frequency of conversation documentation. Similar results were found after the SICP implementation in acute care and internal medicine [9, 10].

Many factors can impact the quality and content of communication between patients and providers [11]. What is not known, is how the content of documentation might vary with patient characteristics and severity of their illness. Exploring this might highlight areas for quality improvement including clinician training, patient preparation for conversations or systemic issues or inequities. Therefore, we sought to explore for associations between the quantity and type of elements documented after SICP conversations in acute care with patient’s goals of care orders and the demographic variables such as age, gender, frailty, understanding of English. Even though the intention of the SICP guide is that all questions are asked, and the answers are documented, we hypothesized that what clinicians choose to document may be dependent on patient characteristics. Particularly, we hypothesized a close association between what is documented and the patient’s goal of care order that communicates their priorities.

## Methods

### Design and setting

This is a secondary study of documentation of Serious Illness Conversations (SIC) collected in a multi-site Canadian quality improvement implementation of the SICP in acute care, internal medicine units [12]. Our site was a 38-bed Medical Teaching Unit at Foothills Medical Centre, Calgary, Canada. SICP implementation and data collection occurred March 2018 to December 2019. Thirty internal medicine physicians (15 female) attended on the unit, with three on service each week. As part of the initiative, each physician aimed to conduct and document at least one SIC per week. Physicians used the structured SIC guide [13] to facilitate discussion of key concepts: patient’s understanding of their health, information preferences and prognosis, goals, fears, strengths, critical abilities, trade-offs they are willing to make for the possibility of more time and family’s understanding of the patient’s wishes. All components of SICP were implemented, including screening to identify appropriate patients, training and cueing clinicians, preparing patients, and documentation of conversations in the electronic health record (EHR).

### Patient selection

Based on local data [14] physicians prioritized having SIC with patients 65 years of age or older who were hospitalized for 5 days or more. This identified about 3–5 seriously ill patients per week per attending. A unit champion (charge nurse) screened the unit list weekly and cued the physicians to consider whether eligible patients would benefit from a SIC. If physicians felt another patient was a higher priority for a SIC conversation, they were able to select patients outside the prioritization criteria. Patients with a previous documented SIC conversation were excluded. Patients accepting of conversations were asked who they would like to be present (e.g., family or friends).

### Conversation documentation

Physicians were trained to document SIC details in the EHR (Sunrise Clinical Manager) and were reminded to document by the unit champion. The conversation document was the “Advance Care Planning (ACP) and Goals of Care Designation (GCD) Tracking Record” (Tracking Record) [15]. It was introduced in 2008 as part of an implementation of a region-wide ACP and GCD policy [16] and procedure [17] as a standard place to record pertinent detailsof conversations and to create a continuous narrative, as conversations are added over time. A copy of the Tracking Record is provided to the patient in a plastic file called the “green sleeve.” This holds and transfers their other ACP documents, such as their medical order (GCD) [18] and Personal Directive [19] (provincially legislated advance directive) across care settings. An anonymized copy (redacted for patient/staff identifiers) of the Tracking Record was retained for this study.

GCDs are a medical order framework [18] that concisely communicate the general focus and indicate the kinds of treatments and locations of care that may best serve the patient’s goals and preferences. There are three general GCD approaches: a) ‘Resuscitative Care’ to cure or control illnesses using life prolonging interventions if clinically required (Resuscitative GCD); b) ‘Medical Care’ to cure or control illnesses, but not including resuscitative or ICU interventions (Medical GCD); and c) ‘Comfort Care’ focusing on a palliative approach by managing symptoms and optimizing function to the degree possible (Comfort GCD).There are subcategories within these approaches, with seven possible GCD orders [18]. Nuances about a GCD or its interpretation for a given patient can be documented in the Tracking Record.

### Evaluation tool

We evaluated the quantity and comprehensiveness of documentation of conversations in the Tracking Record using a standardized SICP codebook [8]. We retained all four domains from the original codebook: (1) Values or goals; (2) Prognosis or illness understanding; (3) End-of-life care planning; (4) Life-sustaining treatment preferences. However, we made one significant update to this record by adding a “Strengths” element and deleting the “Quality of Life” element under the “Goals and Values” domain, to reflect the content of the 2017 SIC guide revision. We also adapted the instructions for the coding to recognize GCD orders in the “Life sustaining treatment preferences” domain (re-titled “GCD and Life sustaining treatment”) (Supplement 1 adapted SICP Codebook). The total possible score remained 17, with a score of “0” (absent) or “1” (present) for each individual element. The higher the total score the more conversation content has been documented.

### Data extraction

All tracking records completed by physicians during SICP implementation were randomly distributed among four raters (JS, SJ, JRS, MD). Raters were two physicians, the unit champion and a researcher, respectively. Copies of the tracking records were anoymised by redacting Identifying data of patients, family or clinicians before distribution. Using the adapted codebook, raters independently abstracted and scored the content of the Tracking Record.

To minimize interpretation and scoring variation, raters met to train on the codebook and collectively code five tracking records. Raters met again to discuss issues or discrepancies after completing coding of 20 tracking records. To verify consistency across raters and calculate inter-rater reliability, a subset of 20 randomly selected tracking records (11%) were coded independently by all raters.

Patient demographic data collected as part of the quality improvement study were: age, gender, frailty scores (summarized using the 8-point version of the Clinical Frailty Score [20]) and ability to understand and speak English as perceived by bedside clinicians (whose conversations were conducted using a language interpretation phone line, or ad hoc family translation, according to patient preference). The latest GCD order in effect on the EHR on the day the conversation occurred was recorded. If the GCD changed that day, after the conversation, that was the GCD recorded.

### Statistical analysis

The primary outcome of interest was the total score (max. 17) for each tracking record. The other outcomes of interest were the subdomain scores (Values or goals; Prognosis or illness understanding; End-of-life care planning; Life-sustaining treatment preferences). For the descriptive statistics, mean and standard deviations were reported for normally distributed continuous variables, median and interquartile ranges were reported for non-normally distributed continuous variables. Frequency and proportions were reported for categorical variables. Generalized linear model (GLM) was used to determine the factors associated with the outcome variables: total score, goals, and “values domain” subtotal, “prognosis domain” subtotal, “end-of- life domain” subtotal and “GCD and life sustaining treatment domain” subtotal. Normal distribution was assumed for the outcome variables and link identity was used for the GLM model. Univariate and multivariate GLM models were analyzed for each outcome variable separately. The factors included in the univariate and multivariate models were gender (Male vs. Female, there were no non-binary identifying patients), able to speak English for the conversation (yes vs. no), age (35–74 years vs. ≥ 75 years, dichotimized at the median age), frailty category (very fit or well or managing well or vulnerable vs. mildly or moderately or severely or very severely frail) and GCD categories (Medical vs. Resuscitative, Comfort vs. Resuscitative). The final model was all adjusted for age, gender, frailty category and ability to speak English as our models adjusted for the most common confounding factors. The final model for all of the outcome variables showed that GCD as an independent variable, indicating that adjusted and unadjusted GCD variables shows significant association with total scores as well as subdomains. All statistical analysis was conducted using SPSS version 25 software [21]. A *p*-value < 0.05 was used for statistical significance.

Intra class correlation tests were conducted to assess the inter-rater variability. The correlation value between raters was 0.726 on single measures, 0.914 on average measures (*p* value < 0.0001, 95% confidence interval, 0.914 reliability statistic). The correlation value > 0.90 represents excellent agreement between the raters. A value between 0.75 to 0.90 represent good measure of agreement [22].

## Results

### Recruitment

**Fig. 1:**
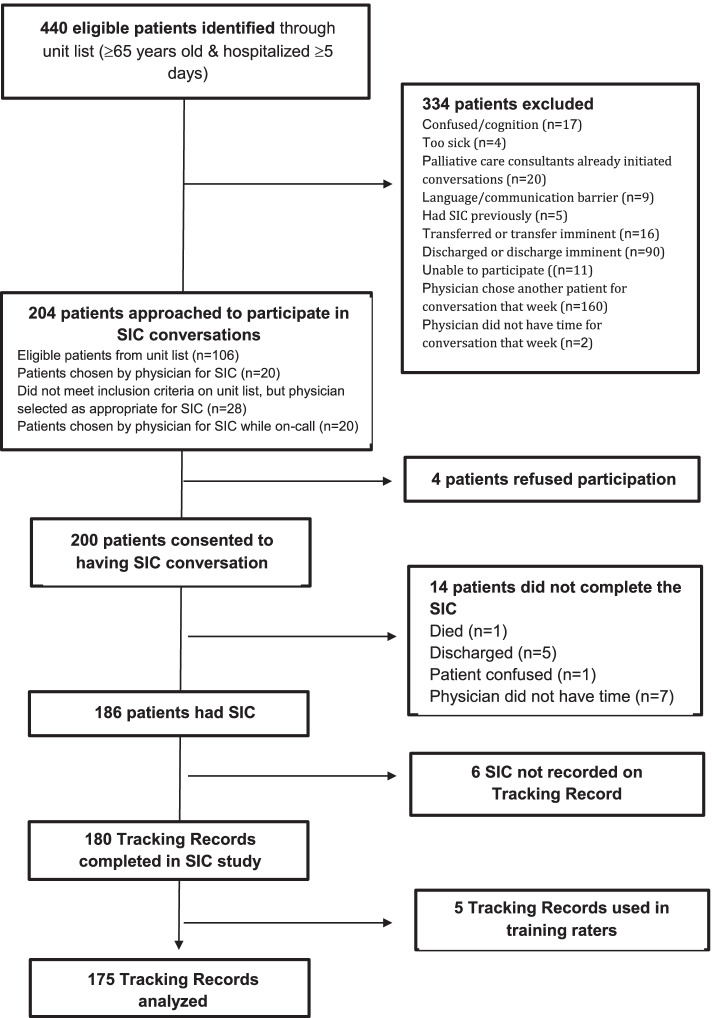
Strobe diagram of patients recruited for Serious Illness Conversations (SIC) and the number of Tracking Records analyzed

Figure 1 shows 440 potential patients were identified by the unit list screening criteria; 78 patients were selected by physicians outside those inclusion criteria, and a further 20 patients had a SIC with their physicians but were not identified through the cueing system (e.g., occurred during weekends without nurse champion present). After all exclusions, refusals and attrition, 180 tracking records of SIC were completed by physicians in the electronic health record and 175 of these were analyzed (5 used in codebook training).

### Patient characteristics

Patients’ demographic characteristics in Table 1 shows fewer tracking records were from conversations with female patients (69 (39.4%) vs. 106 (60.6%)). The mean age of patients was 73.8 years (SD = 11.2) and majority (65.1%) of tracking records were for patients rated as vulnerable (category 4) or mildly frail (category 5) on Clinical Frailty Scale, and patients understood/spoke English (88%). Of the three GCD categories, the majority of tracking records were also for those patients that had a Medical GCD (43.4%).

**Table 1:** Characteristics of patients who had a Serious Illness Conversation documented on Tracking Record as part of the Serious Illness Conversation Program implementation

	**No. (%)**
**Characteristic**	***n* = 175**
Female	69 (39.4)
Age	
32–74	84 (48.0)
≥ 75	91 (52.0)
Clinical Frailty Score	
Very Fit (Category 1)	3 (1.7)
Well (Category 2)	10 (5.7)
Managing well (Category 3)	30 (11.4)
Vulnerable (Category 4)	52 (29.7)
Mildly Frail (Category 5)	62 (35.4)
Moderately Frail (Category 6)	16 (9.1)
Severely Frail (Category 7)	12 (6.9)
Very Severely Frail (Category 8)	0 (0)
Speak or understand English	154 (88)
GCD	
R (resuscitative)	51 (29.1)
M (medical)	76 (43.4)
C (comfort)	48 (27.4)

### Comprehensiveness of conversations

**Fig. 2:**
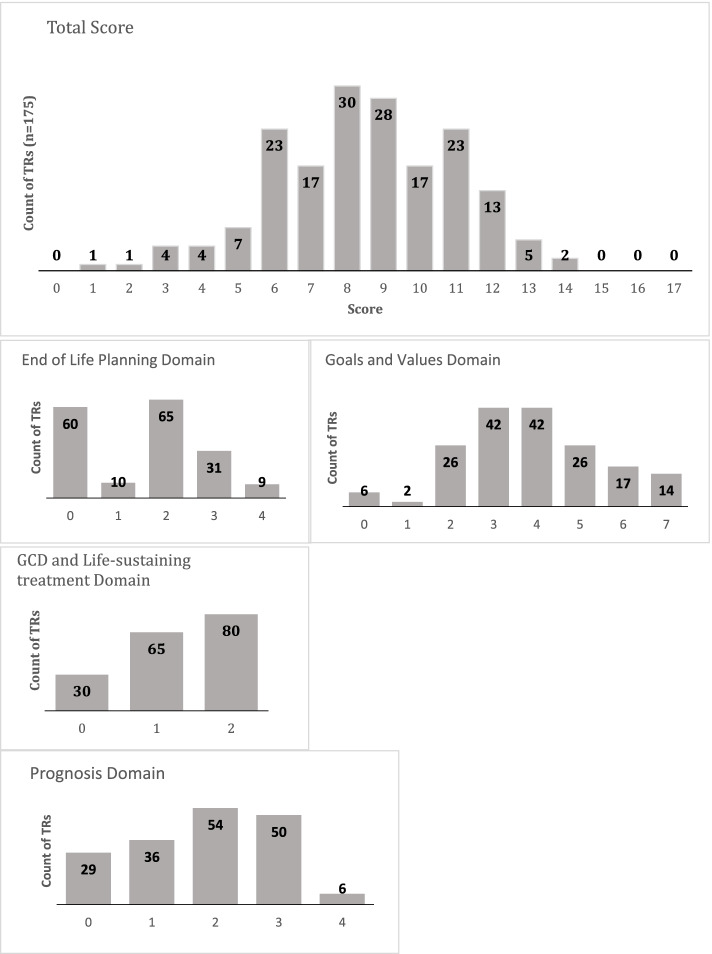
Distribution of documented Serious Illness Conversation element scores (total score and by domain) for Tracking Records. Tracking Records for each patient (*n* = 175) were scored on whether elements from the Serious Illness Conversation Program Codebook [8] were present (1) or absent (0). The Total score (0–17) was comprised of the total score in each domain: Goals and Values (0–7), Prognosis (0–4), End-of-Life Care Planning (0–4), and Goals of Care Designation/Life-sustaining treatments (0–2)

The median total score of conversation elements documented on the tracking records was 9 (IQR 7- 10, Fig. 2) with the fewest (65.7%) tracking records documenting end-of-life care domain content and most (96.6%) documenting at least one Goal or Value content.

### Associations

In the univariate analysis (Table 2), there was no statistically significant difference between total or domain scores by gender, age, or frailty. More goals and values were documented for patients who were able to conduct the conversation in English (0.89; 95% CI: 0.14–1.63). More total content was recorded for patients who had a Medical or Comfort GCD than for Resuscitative GCD (“Medical”: 2.42; 95% CI: 1.51 – 3.33; “Comfort”: 1.06; 95% CI: 0.24 – 1.88).

**Table 2:** Univariate analysis of patient characteristics (age, gender, Clinical Frailty Score, ability to understand/speak English and Goals of Care Designation (GCD)) and Serious Illness Conversation documentation element scores according to Serious Illness Conversation Program Codebook

	Total Scores		Goals and Values		Prognosis		End of Life		GCD and Life sustaining treatment	
	Median	Estimate (95% CI)	Median	Estimate (95% CI)	Median	Estimate (95% CI)	Median	Estimate (95% CI)	Median	Estimate (95% CI)
**Age**										
< 75 years	8.00	-0.52 (-1.26; 0.21)	4.00	-0.42 (-0.91; 0.06)	2.00	0.15 (-0.19; 0.48)	2.00	-0.07 (-0.45; 0.30)	1.00	-0.14 (-0.36; 0.08)
≥ 75 years	9.00	Ref	4.00	Ref	2.00	Ref	2.00	Ref	1.00	Ref
**Gender**										
Male	9.00	0.20 (-0.56; 0.95)	4.00	-0.04 (-0.54; 0.46)	2.00	0.15 (-0.19; 0.49)	2.00	-0.05 (-0.43; 0.34)	1.00	0.09 (-0.14; 0.31)
Female	8.00	Ref	4.00	Ref	2.00	Ref	2.00	Ref	1.00	Ref
**Frailty**^a^										
Not Frail	8.00	-0.12 (-0.86; 0.62)	4.00	0.47 (-0.01; 0.96)	2.00	-0.24 (-0.57; 0.09)	2.00	-0.18 (-0.55; 0.20)	1.00	-0.12 (-0.34; 0.10
Frail	9.00	Ref	3.00	Ref	2.00	Ref	2.00	Ref	1.00	Ref
**Language**^b^										
English	9.00	0.35 (-0.79; 1.48)	4.00	0.89 (0.14; 1.63)*	2.00	-0.15 (-0.67; 0.36)	2.00	-0.31 (-0.88; 0.27)	1.00	-0.05 (-0.39; 0.28)
Non-English	8.00	Ref	3.00	Ref	2.00	Ref	2.00	Ref	1.00	Ref
**GCD**										
Resuscitative	7.00	Ref	4.00	Ref	1.00	Ref	0.00	Ref	1.00	Ref
Medical	8.00	2.42 (1.51; 3.33)*	4.00	-0.82 (-1.45; -0.18)*	2.00	1.02 (0.61; 1.44)*	2.00	2.00 (1.60; 2.40)*	2.00	0.28 (-0.01;0.56)
Comfort	10.00	1.06 (0.24; 1.88)*	3.50	-0.45 (-1.03; 0.12)	2.00	0.47 (0.10; 0.84)*	3.00	0.74 (0.38; 1.10)*	1.00	0.30 (0.05;0.56)*

^a^Clinical Frailty score 1–3 (not frail) Vs. 4–8 (Frail)l

^b^English understood and spoken by patient Vs. English not understood and spoken by patient

^*^p < 0.05; Ref = Reference category

**Fig. 3:**
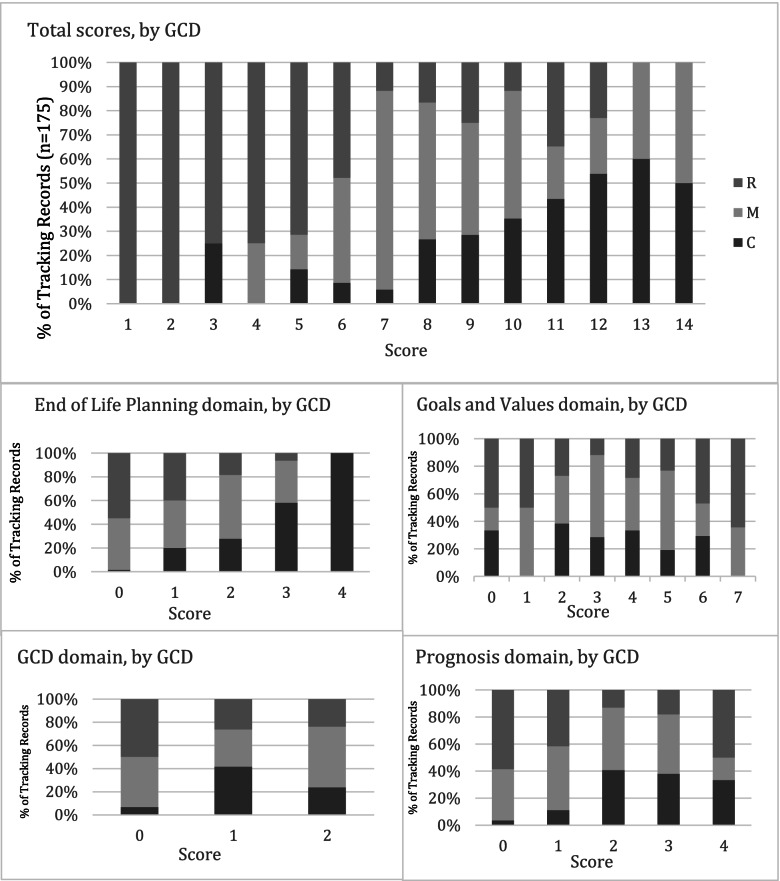
Percentage of Tracking Records recording Serious Illness Conversation element scores (total score and by domain) by patient’s Goals of Care Designation. Total and domain scores of elements from the Serious Illness Conversation Program Codebook [8] for Tracking Records (*n* = 175). Elements were coded as 1 if present and 0 if absent. The Total score (0–17) was comprised of the total score in each domain: Goals and Values (0–7), Prognosis (0–4), End-of-Life Care Planning (0–4), and Goals of Care Designation/Life-sustaining treatments (0–2). Distributions shown, are by patient’s Goals of Care Designation (GCD) which are a) ‘Resuscitative Care’ (R); b) ‘Medical Care’ (M); and c) ‘Comfort Care’ (C)

In the multivariate analysis, controlling for age, gender, language and frailty, the association between total Tracking Record scores and GCD remained highly significant (Table 3). Specifically, Tracking Records completed for patients with a Comfort or Medical GCD had higher total scores (“Comfort”: 2.667; 1.710 – 3.624; “Medical”: 1.274; 0.418 – 2.130) than those completed for patients with a Resuscitative GCD (Fig. 3). Similarly, scores were positively associated with a Comfort GCD and Medical GCD in the prognosis (“Comfort”: 1.067; 95% CI: 0.630–1.503; “Medical”: 0.545; 95% CI: 0.154–0.936) and end-of-life planning (“Comfort”: 2.132; 95% CI: 1.714–2.551; “Medical”: 0.807; 95% CI: 0.433–1.182) domains. Conversely a *negative* association was found between the Goals and Values domain score and patients with a Comfort GCD (-0.687; 95% CI: -1.346—-0.027). For the GCD and life sustaining therapy domain, patients with a Medical GCD had significantly higher scores compared to reference category Resuscitative GCD (0.327; 95% CI: 0.057–0.598), but not Comfort versus Resuscitative (0.251; 95% CI: -0.051–0.553). In the multivariate model none of the other variables were associated with the total or domain scores with one exception: there was a statistically significant positive association between the score in the goals and values domain and age > 75 years (β = 0.586; 95% CI: 0.107—1.065).

**Table 3:** Results of the generalized linear model (adjusted for age, gender, frailty, speaking English language) showing the association between Goals of Care Designation (GCD) and Tracking Record total and individual domain scores

	B	Std Error	Lower CI	Upper CI	Wald Chi-Squared	*P* -value
Total Score						
C^a^ Vs. R^b^	2.667	0.488	1.710	3.624	29.85	** < 0.001**
M^c^ Vs. R	1.274	0.437	0.418	2.130	8.506	**0.004**
Goals and values domain						
C Vs. R	-0.687	0.3365	-1.346	-0.027	4.167	**0.041**
M Vs. R	-0.399	0.3012	-0.990	0.191	1.758	0.185
Prognosis domain						
C Vs. R	1.067	0.223	0.630	1.503	22.950	** < 0.001**
M Vs. R	0.545	0.199	0.154	0.936	7.476	**0.006**
End-of-life Domain						
C Vs. R	2.132	0.213	1.714	2.551	99.915	** < 0.001**
M Vs. R	0.807	0.190	0.433	1.182	17.880	** < 0.001**
GCD and life-sustaining therapy domain						
C Vs. R	0.251	0.154	-0.051	0.553	2.652	0.103
M Vs. R	0.327	0.138	0.057	0.598	5.634	**0.018**

^a^Comfort, ^b^Resuscitative, ^c^Medical

## Discussion

This study is novel in exploring and finding associations between the quantity and type of conversation elements documented by physicians after SIC conversations and the patient’s GCD (the medical order communicating a focus on comfort, medical or resuscitative care). For patients with a Comfort or Medical GCD, physicians recorded more fulsome conversations with higher total scores and more elements documented in two domains: prognosis, end-of-life planning, than for patients with a Resuscitative GCD. In contrast, physicians were less likely to comprehensively document patient’s goals and values with patients with a Comfort GCD. It has not been previously reported whether the quality of documentation after SIC is associated with other frameworks like Physician orders for Life Sustaining Therapy (POLST) [23], or “Do-Not- Attempt-Resuscitation” orders.

This association between patient GCD and the total scores and sub-domain content documented is unsurprising and may be understood by reflecting on clinical practice. When a patient is coming closer to end-of-life and the goal of care is determined to be on comfort more than prolonging time, physicians may naturally focus documentation on what they perceive to be the most relevant elements and domains, such as discussion about hospice care (End-of-Life care domain), or prognostic communication about worsening of disease (Prognosis or Illness understanding). In contrast when documenting conversations for patients with medical or resuscitative goals of care, physicians may record more information about goals and values because they deem that to be most relevant to healthcare providers during subsequent treatment decision-making and consent discussions. The End-of-life care domain for patients with Medical or Resuscitative GCDs might be less documented because these were less frequently elicited with these patients or are deemed less relevant to document within the patient’s current priorities or clinical context. We note clinicians are not *specifically* trained or prompted to explore or document about end-of-life planning using the SIC guide although this is a domain in the codebook. Nonetheless, physicians documented about the end-of-life planning domain in 66% of conversations. It is somewhat worrying, that patients with resuscitative goals had less total content documented. This could represent a physician bias towards less fulsome conversations or documentation about the use of life sustaining therapies with these patients.

There is also an ongoing concern about equity and inclusion in advance care planning [24]. We found gender, age and frailty were not associated with differences in documentation content, however fewer patients’ goals and values were documented for patients who were unable to conduct the conversation in English. Only 21 such patients were included in the study and of note nine patients who met inclusion criteria were not selected by physicians for conversations because of “language/communication barriers.” Although there are multiple language SIC translations, its use requires the medical interpreter to have direct access to the appropriate version. Our centre’s lack of in-person interpretation may limit a physician’s ability to directly elicit patients’ own values, as these are conveyed through a phone-based language translation line or a family member. It is not known what was ‘lost in translation’ or what physician or family biases were factors in what was discussed or documented. Indeed, patient selection for conversations was up to the discretion of the physician and therefore open to potential physician selection biases including gender and language ability. The reasons behind these differences, and exploring other intersecting equity factors such as ethnicity, socioeconomic status are starting points for further research.

A study strength included using the standardized codebook, to allow comparison with other SICP studies. Our median score of 9 conversation elements recorded on the tracking record was identical to the median score for conversations documented using a templated letter format at another acute care site [10]. Other studies [8–10] have not reported the inter-rater agreement when applying the codebook and this is of concern because although there was high agreement for average rating scores, inter-rater agreement on single measures was only moderate.

In addition to the major limitation of selection bias in who was selected or excluded for a SIC conversation, another limitation is that we did not analyze who was present for the SIC conversations (e.g., family, friends, substitute decision makers) or how language translation was provided. It was also difficult to collect how many GCDs may have changed because of the SIC conversation, as GCD changes can occur as part of a process of conversations and reflection happening over a few days or weeks. We are also unable to assess the gap between what was *actually* discussed and what was subsequently documented in health records. There may also be a physician bias towards documenting conversation elements that support or align with the patient’s exisiting GCD or the GCD determined through the SIC converstion. Geerse et al. [25] compared cancer outpatient audiotaped SIC conversations and documentation and found that 36% of conversational information was not documented; with function, fears and worries and tradeoffs most often discussed but not documented. They found high (87%) adherence with asking about SIC guide components but only 43% of all conversation elements were deemed fully concordant with their documentation.

## Conclusion

This study is the first to demonstrate that the quantity and type of conversation domains documented by physicians after a SIC conversation varies with the medical orders describing the patient’s goals of care. How this varied documentation impacts subsequent clinical practice is a focus for our future studies. Other factors like age, gender, frailty, were not as significantly associated with the quality of conversation documentation but the findings point towards a need to attend to language barriers in eliciting patients own goals and values. The practice implications are that clininicians conducting and documenting SIC conversations should be aware of potential personal and systemic biases when eliciting patients’ priorties and actively listen and accurately document what is expressed.

## Supplementary Information

**Additional file 1: Supplement 1.** Revised Codebook rules for coding serious illness conversations on the tracking record.

## Data Availability

The datasets used and/or analyzed during the current study are available from the corresponding author on reasonable request.

## Abbreviations

SICP: Serious Illness Care Program
SIC: Serious Illness Conversations
EHR: Electronic health record
ACP: Advance Care Planning
GCD: Goals of Care Designation
Resuscitative GCD: Resuscitative Care Goals of Care Designation
Medical GCD: Medical Care Goals of Care Designation
Comfort GCD: Comfort Care Goals of Care Designation
GLM: Generalized Linear Model
