# Supplementary material for: Content of Serious Illness Care conversation documentation is associated with goals of care orders—a quantitative evaluation in hospital
Source: BMC Palliat Care. 2022 Jun 29;21:116. doi: 10.1186/s12904-022-01006-2 (PMC9241276; doi:10.1186/s12904-022-01006-2)
Supplement: Supplementary file 1 — Additional file 1: Supplement 1. Revised Codebook rules for coding serious illness conversations on the tracking record. [file 12904_2022_1006_MOESM1_ESM.docx]

**Supplement 1:** Revised Codebook rules for coding serious illness conversations on the tracking record.

| **GOALS AND VALUES**  *7 total items*  *For scoring purposes – Goals of care can be EITHER specific OR non-specific* | |
| --- | --- |
| Subdomain | Description and Examples |
| Goals of care (unspecified) | Documentation of a discussion about “goals” or “goals of care”, without mention of specific goals or priorities. Includes set-up of serious illness conversation.  Examples: “We also discussed goals of care”; “30 minutes were spent discussing goals of care”  “I explained to him that this is a chance for him and for us to reflect on his health status and think about the future.”    If any one or more of the following subdomains were addressed, a point was also given in this subdomain.^[[1]](#footnote-1)^ |
| Goals, priorities, “is important to” (specific) | Documentation of a discussion about particular goals important to the patient, excluding “treatment decisions” (e.g., decisions about chemotherapy regimens).  Examples: “The patient wants to be at home/live as long as possible/not be a burden”; “His priority is to be able to teach his courses this fall”; “It is important to her to be able to enjoy their trip to Hawaii in the spring” |
| Fears, worries | Documentation of a patient’s fears, worries or concerns  Example: “He worries about becoming dependent and ‘dying without dignity’” |
| Sources of strength | Documentation of what gives a patient strength or support as the patient thinks about the future with their illness.  Example: Her religion (Catholicism) is a source of strength. |
| Tradeoffs | Documentation of what a patient is willing to go through (e.g., for the possibility of more time)  Example: “He does not want to experience any major side effects unless there is a high likelihood of therapeutic benefit” |
| Function, abilities | Documentation of abilities that are critical to the patient  Example: “Maintaining his ability to interact with others is important to him” |
|  |  |
| Family involvement/Support for Family/ADM | Documentation of how much family/ADM knows about the patient’s priorities/wishes; how much the patient wants family to be involved in further decisions; planning for family to be present at subsequent discussions; the role of the family in the patient’s care or how the family is affected by the patient’s illness.  Examples: “We talked about how he and his wife might begin to have conversations with their daughters.”; “We talked about how her son has been helping her manage at home.” “We talked about how she feels she is a burden on her son and how he needs more support as a caregiver.”    A mention that family was present for the conversation do not count as a point.  Example: “Patient’s sister present for meeting” |
| **PROGNOSIS OR PROGNOSTIC UNDERSTANDING**  *4 total items*  *For scoring purposes – Prognosis is EITHER specific OR non-specific* | |
| Information preferences | Documentation of the patient’s preferences to receive information about prognosis or the future.  Example: “Patient stated she would like to receive prognostic information frequently and in the presence of family” |
| Prognostic understanding | Documentation of the patient’s understanding of illness or prognosis  Example: “We talked about his cancer today, and he understands that his tumor is incurable”; “He knows he only has weeks to live” |
| Prognosis/life expectancy (unspecified) | Documentation of a discussion about prognosis or life expectancy, without specific communication of time, function, or QOL.  Example: “We discussed his prognosis today”; “30 minutes were spent today answering their questions about prognosis and treatment options”    If the following subdomain was addressed, a point was also given in this subdomain.^[[2]](#footnote-2)^ |
| Prognostic communication about time, function, or QOL; no more treatment options, progression of disease, worsening of disease, functional decline  (specific) | Documentation of specific mentions of prognosis (in terms of time, function, or QOL) or discussion of no more treatment options, progression of disease, worsening of disease, functional decline  Example: “They had questions about prognosis. I shared that he likely has weeks to months left.”; “We discussed this is likely the best the patient will feel, and the disease will cause worsening decline” |
| **END-OF-LIFE CARE PLANNING**  *4 total items* | |
| End of life, end of life planning, EOL, advance care planning (unspecified) | Documentation of any of these keywords in the context of a broader discussion. Example: “Today we discussed the patient’s end-of-life preferences.”    If any one or more of the following subdomains were addressed, a point was also given in this subdomain.^[[3]](#footnote-3)^ |
| Palliative care, supportive care, comfort-focused care | Documentation of discussion about future use of, initiating or transitioning to palliative care, supportive care, or comfort-focused care (not including palliative chemotherapy).  Example: “She and her family indicated that given the circumstances, they would like to start a comfort-oriented approach.”    Report of use of palliative approach to care alone did not count without documentation of a discussion.  Example: “Palliative care service following for symptoms management” |
| Hospice | Documentation of any of these keywords in a discussion.  Example: “After a lengthy discussion, we have opted to discontinue therapy and proceed with referral to hospice.” |
| Site of death/Practical planning | Documentation of a discussion indicating where the patient wants to be at death (e.g., at home, or at hospice), medical assistance in dying (“MAID”), or about estate planning or legal documents.  Example: “Patient wishes to die at home” |
| **Goals of Care Designation OR LIFE-SUSTAINING TREATMENTS**  *2 total items* | |
| Specific Goals of Care Designation | Documentation of discussion with keywords “GCD”, “R”, “M”, “C”, “R1”, “R2”, “R3”, “M1”, “M2”, “M3”, “C1” or “C2”  Example: “At this point he wishes to remain R1 goals of care . . .”    Reports of GCD alone do not count without documentation that a discussion occurred. |
| Life-sustaining treatments (Also: chest compressions/ intubation/shocks/ feeding tube/ICU) | Discussion specifying life sustaining treatments that are within patients desired scope of care.  Examples: “We talked about whether the patient would want CPR if her heart stopped beating” |

Adapted from Lakin, Koritsansky et al.^8^

1. Further explanation/clarification: whenever a point is awarded for **any one or more** of the following (specific) 7 “Goals and Values” **subdomains**: 1. Goals, priorities, “is important to” (specific); 2. Fears, worries; 3. Sources of strength; 4. Tradeoffs; 5. Functions, abilities; 6. Family involvement/Support for Family; a point is **also** given in the (general) subdomain “Goals of care (unspecified)”. [↑](#footnote-ref-1)
2. Further explanation/clarification: whenever a point is awarded for the (specific) subdomain “Prognostic communication about time, function, or QOL; no more treatment options, progression of disease, worsening of disease, functional decline (specific)”, a point is **also** awarded in the (general) “Prognosis/life expectancy (unspecified)” subdomain. [↑](#footnote-ref-2)
3. Further explanation/clarification: whenever a point is awarded for **any one or more** of the following 3 (specific) subdomains: 1. Palliative care, supportive care, comfort-focused care; 2. Hospice; 3. Site of death/Practical planning; a point is also awarded in the (general) “End of life, end of life planning, EOL, advance care planning (unspecified)” subdomain. [↑](#footnote-ref-3)
